# Construction and Maintenance of Building Geometric Digital Twins: State of the Art Review

**DOI:** 10.3390/s23094382

**Published:** 2023-04-28

**Authors:** Viktor Drobnyi, Zhiqi Hu, Yasmin Fathy, Ioannis Brilakis

**Affiliations:** Department of Engineering, University of Cambridge, Cambridge CB3 0FA, UK; vd312@cam.ac.uk (V.D.); yafa2@cam.ac.uk (Y.F.); ib340@cam.ac.uk (I.B.)

**Keywords:** digital twins, geometric digital twins, building information modelling, object detection, object segmentation, scan-to-BIM, scan-vs-BIM, deep learning, construction of digital twins, maintenance of digital twins

## Abstract

Most of the buildings that exist today were built based on 2D drawings. Building information models that represent design-stage product information have become prevalent in the second decade of the 21st century. Still, it will take many decades before such models become the norm for all existing buildings. In the meantime, the building industry lacks the tools to leverage the benefits of digital information management for construction, operation, and renovation. To this end, this paper reviews the state-of-the-art practice and research for constructing (generating) and maintaining (updating) geometric digital twins. This paper also highlights the key limitations preventing current research from being adopted in practice and derives a new geometry-based object class hierarchy that mainly focuses on the geometric properties of building objects, in contrast to widely used existing object categorisations that are mainly function-oriented. We argue that this new class hierarchy can serve as the main building block for prioritising the automation of the most frequently used object classes for geometric digital twin construction and maintenance. We also draw novel insights into the limitations of current methods and uncover further research directions to tackle these problems. Specifically, we believe that adapting deep learning methods can increase the robustness of object detection and segmentation of various types; involving design intents can achieve a high resolution of model construction and maintenance; using images as a complementary input can help to detect transparent and specular objects; and combining synthetic data for algorithm training can overcome the lack of real labelled datasets.

## 1. Introduction

This paper provides a comprehensive review of the methods for constructing and maintaining the as-is geometry of a building digital twin (DT). A building refers to the structure comprised of connected object instances (e.g., walls, roofs, beams, columns, windows, doors, etc.) along with mechanical, electrical and plumbing (MEP) systems (e.g., piping and duct systems, fire protection systems, etc.). Geometry refers to three-dimensional (3D) objects and their relationships. A DT serves as a product and process information repository for storing and sharing physical and functional properties of a building over time with architectural, engineering, construction, and operation (AECO) stakeholders throughout its lifecycle [1]. The construction of as-is geometric digital twins (gDTs) refers to generating building geometry as defined above for existing buildings during their operation stage without prior building geometry information from point cloud datasets (PCDs). On the other hand, the maintenance of geometric digital twins refers to updating building geometry with the help of existing building information models (BIMs) representing the design intent (DI). Maintenance aims to keep gDTs up-to-date during the whole lifecycle of buildings, primarily during the construction and operation stages. The construction and maintenance of gDT together aim to obtain the building’s geometry as product information at different timestamps throughout the building’s lifecycle, thus facilitating the more efficient planning, construction, and operation of buildings. The main problem for unlocking the practical value of gDTs is that the costs and time of digitising the geometry of buildings still outweigh the benefits of DTs. This limits the adoption of digital technologies at every stage of the building lifecycle for new and existing facilities. The automation of digitalisation can help improve the efficiency of operations, reduce the cost and schedule overrun, and increase productivity [2]. The AECO industry remains one of the least digitised sectors of the global economy [3], which causes its low productivity. McKinsey Global Institute also shows that the economy loses about USD 1.6 billion every year due to the low digitisation of the construction sector [2].

The construction of gDTs aims to provide product information for existing buildings, since they lack digital representations to support efficient operation and renovation. This problem is worse for old buildings because their as-designed product information still sits in two-dimensional (2D) drawings that are often inaccurate or outdated over time. More than half of the buildings in service in the UK were built more than 20 years ago [4,5]. This explains why their design models are not available in a digital format [6] or are not reliable representations of current as-is geometry [7]. Around 85–95% of existing buildings will remain in use until 2050 [8] and many will need to be renovated. Owners and managers of such buildings cannot fully benefit from the 2D drawings mentioned above to assist buildings’ operation and renovation.

**Figure 1:**
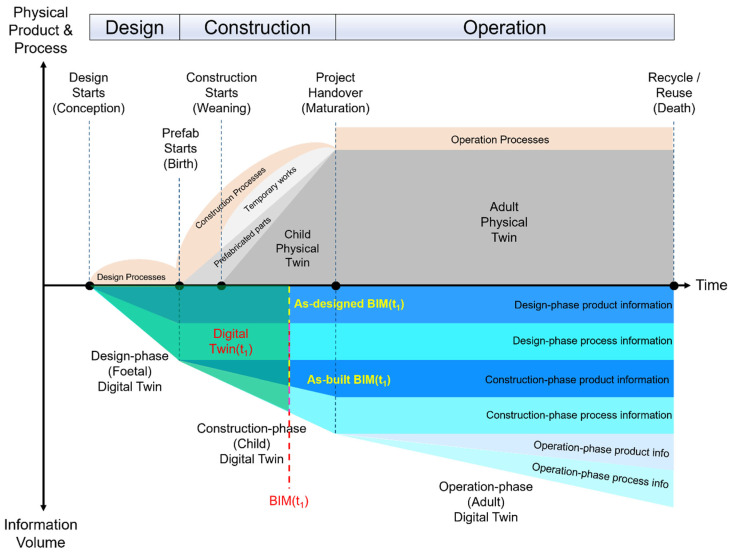
Lifecycle of the physical and digital building twins with product and process information (updated from Sacks et al. [1]).

On the other hand, the maintenance of gDTs aims to provide dynamic, updated product information for new buildings in the construction stage to support progress monitoring and quality control. Maintaining gDTs requires a DI model (usually referring to the final as-designed model) serving as a benchmark to the assist updating process. Figure 1 shows a typical building’s lifecycle of a physical twin and its corresponding DT from the design and construction to the operation stage. Specifically, the foetal DT at the design stage contains both product and process information, where the product information on the various timestamps refers to as-designed BIMs. The client-approved final design file at the end of timeline in the design stage is marked as DI, which means it will serve as the benchmark for evaluating the construction outcomes. As-built product information is collected on a timely basis and accumulated until the completion of the construction to deliver a child DT during the construction stage. gDT needs to be updated regularly and iteratively to support progress monitoring and quality control. Finally, the adult DT can support building performance analyses for energy consumption and building components maintenance during the operation stage. Overall, the automatic construction and maintenance of gDTs for buildings is important. This paper mainly focuses on the construction of gDTs for existing buildings at the operation stage and the maintenance of gDTs for buildings at the construction stage. The current state-of-the-art geometry generation and updating methods relying on PCD are still time-consuming and manual-intensive and not reliable and robust in real, complex environments. On this basis, we argue that automating the construction and maintenance of gDTs for buildings is crucial.

This review paper is exploratory and aims to understand the problem of digitising building geometry. Most previous literature review papers have focused on the concept and functions of DT [9,10], applications in construction workforce safety [11], smart manufacturing [12], supply chains [13], or target city scale DTs [14]. Other papers that discuss the geometry of DTs of buildings, such as [15], do not concentrate on 3D geometry processing or lack up-to-date comprehensive recent works in this domain [16]. In this paper, we discuss the state of practice and state-of-the-art methods to enable creating and updating efficient gDTs by relying on PCD, reference models (e.g., an as-designed model), or both. The second objective is to understand to what extent they solve the problem. This implies understanding the scope of the problem where the type and frequencies of objects in a building are unknown. This is a fundamental requirement for generation and maintenance and to understand what methods can detect these object types. We created a geometry-oriented object type categorisation for buildings and established the most frequent object types by analysing a number of IFC files of different buildings to bridge the second knowledge gap in this field. We also excluded heritage buildings from scope of this paper because they constitute a small proportion of all buildings and their geometry is dissimilar to other constructions.

The main contribution of this paper is to (1) investigate the state-of-the-art practice and methods in the research of the automatic generation and maintenance of gDTs; (2) create a geometry-based object class hierarchy; and (3) identify the most frequent building object classes. We analyse existing studies from the perspective of floor and space detection, object detection, object matching, and object comparison in both traditional computer vision and advanced deep learning methods. We summarise the properties, experiments, and limitations of the state-of-the-art methods to reduce knowledge gaps in this area of research. In order to help researchers understand the problem more clearly from a geometric perspective, we created a new geometry-based object class hierarchy that particularly focuses on the geometric properties of building objects, in contrast to widely used existing object categorisations that are mainly function-oriented. The new hierarchy serves as a basic geometry-oriented object classification to assist in determining the most frequently used object classes in buildings. We ranked the frequency of building objects by collecting and analysing IFC models of various building types (e.g., school, hospital, residence, apartment, office, etc.) and identified the top 10 to 14 object classes, covering 75% to 92% of all objects in a building on average. We argue that automating the construction and maintenance of these objects will significantly save time and reduce the cost of adopting DTs for existing and new buildings.

The paper is structured as follows. Section 2 discusses, evaluates, and summarises the state-of-the-art software for generating and updating gDTs for buildings. Section 3 elaborates on the state of research in generating and updating gDTs for buildings as well as related areas of research, including the advantages and limitations of the methods. Section 4 then introduces the new geometry-based object hierarchy and ranks object classes based on their frequency of appearance. Section 5 summarises the state of practice and the state of research with respect to the most frequent object classes and finds gaps in knowledge as well as making suggestions for future research to fill these gaps. Section 6 concludes the paper by summarising the implications for the industry and broader society.

The following search terms were used to collect and investigate the sources and references for this paper.

- Google Scholar: (intitle: ”digital twins” OR intitle: “building information model” OR intitle “geometric digital twins” OR intitle “scan-to-BIM” OR intitle “scan-vs-BIM”) AND (“machine learning” OR “deep learning” OR “construction” OR “maintenance” OR “operation” OR “object detection” OR “object segmentation” OR “instance segmentation” OR “semantic segmentation” OR “geometry updating” OR “geometry generation”).
- Scopus: (TITLE (“digital twins” OR “building information model” OR “geometric digital twins” OR “scan-to-BIM” OR “scan-vs-BIM”) AND TITLE-ABS_KEY (“machine learning” OR “deep learning” OR “construction stage” OR “maintenance stage” OR “operation stage” OR “object detection” OR “object segmentation” OR “instance segmentation” OR “semantic segmentation” OR “geometry updating” OR “geometry generation”)).
- Other websites including “Web of Science”, “iDiscover Cambridge” “GOV.UK” “Mckinsey Global Institute” with the same keywords.

We summarised all results and selected more than 100 papers directly related to this review topic and provided a comprehensive systematic review based on the investigation results.

## 2. State of Practice

This section reviews and assesses available software for the automatic and semi-automatic geometry processing of PCDs for buildings. It first discusses object detection in PCDs in terms of generating the geometry of existing buildings, then covers software assessment related to the maintenance of gDTs that compares PCDs with reference models. The software selection was limited to those designed to work with PCDs and provide tools to extract information from PCDs to assist a modeller. We exclude the software used for the building design from the scope of this paper because it provided only manual tools.

### 2.1. Construction of Digital Twins

Software for constructing gDT from PCDs aims to reduce the manual effort necessary to process PCDs. They perform part of the process automatically or semi-automatically by providing tools for shape detection, object isolation in PCD, and object fitting to PCD using object catalogues. Examples of these software include “EdgeWise”, “Faro as-built modeller”, “Pointfuse”, “Scantobim.xyz”, “InfiPoints”, “Point Cab”, “Vision Lidar”, “Leica Cyclone Model”, “Leica Cloudworx”, “E3D Design”, and “Trimble Realworks”, among others. Some of the software listed above is designed for industrial assets and, therefore, has richer functionality for piping networks and steel structures because they are more commonly used for this type of assets [17].

**Figure 2:**
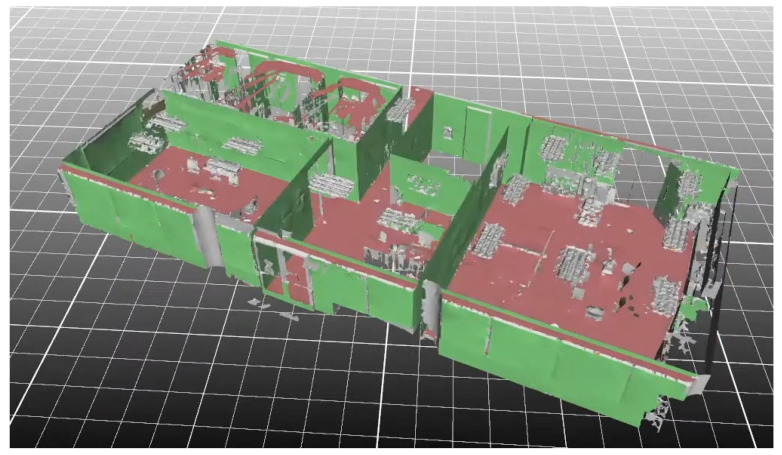
Automatic wall, floor detection in PointFuse. Source [18].

**Figure 3:**
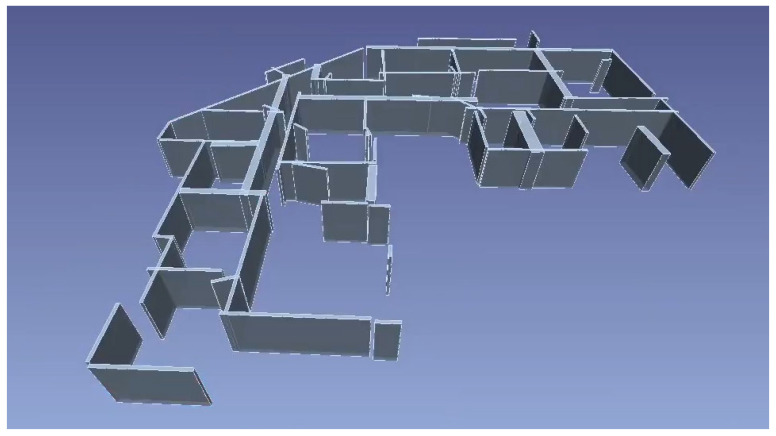
Automatic wall detection in EdgeWise. Source [19].

Some software can automatically detect planar patches in PCDs to generate walls and slabs. For example, “Pointfuse” automatically extracts planar surfaces in PCDs and classifies them based on the normal orientations of these planes: vertical surfaces are considered to be wall segments and horizontal surfaces are ceilings or floors (Figure 2). However, human intervention is required for manually combining and introducing surfaces to generate volumetric models of walls. Alternatively, “EdgeWise” automatically combines detected vertical planes into 3D parallelepipedal models of walls (Figure 3). However, the set of detected walls is incomplete and contains the wrong cuboids. Moreover, the accuracy of wall dimensions is not perfect and requires manually the adjustment of the length of walls. These examples highlight how the manual effort for wall and slab generation is reduced by providing the surfaces or coarse parametric models of these objects.

**Figure 4:**
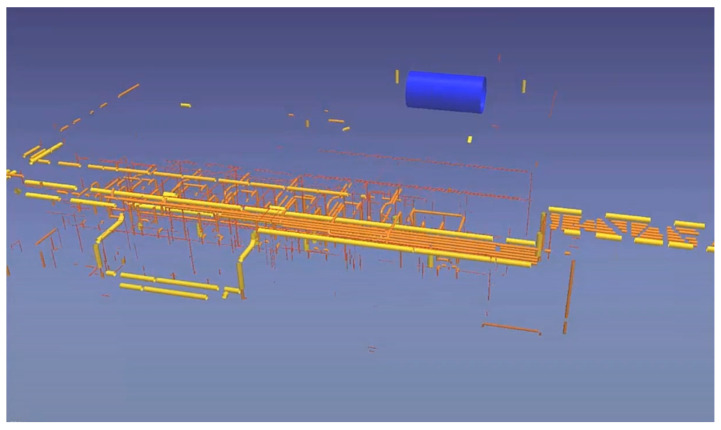
Automatic pipe detection in EdgeWise. Source [20].

**Figure 5:**
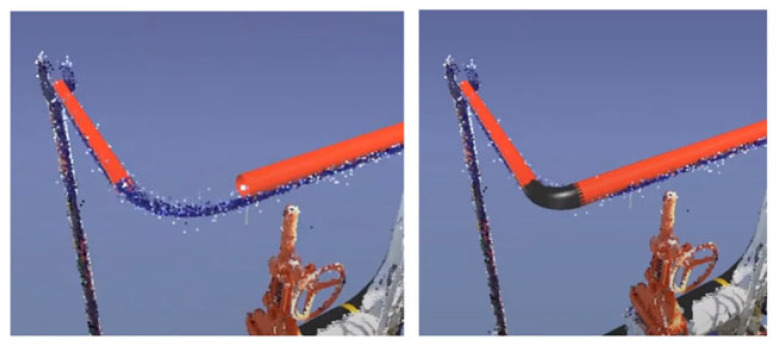
Automatic elbow generation in EdgeWise. Pipe fittings are generated based on pipe endings. Source [21].

A similar approach is used to generate piping elements automatically. For instance, “EdgeWise” assists in generating piping networks by detecting and fitting pipes in a PCD and proposing pipe fittings on pipe endings. This software automatically extracts cylindrical surfaces in a PCD and fits a cylinder as a model of a pipe (Figure 4). A quantitative study shows that EdgeWise achieves 62% precision and 75% recall in detecting cylinders [17]—meaning that three-quarters of pipes can be automatically generated without user intervention. If a pipe is not automatically detected, “EdgeWise” allows the user to isolate a PCD cluster that contains a pipe and automatically fits a pipe there. Lastly, pipe fittings, such as elbows and tees, are localised on pipe endings (Figure 5). Although these fittings introduce connected systems for an asset, their resulting models notably deviate from PCDs. These tools significantly reduce manual effort but leave a notable amount of work for the user.

**Figure 6:**
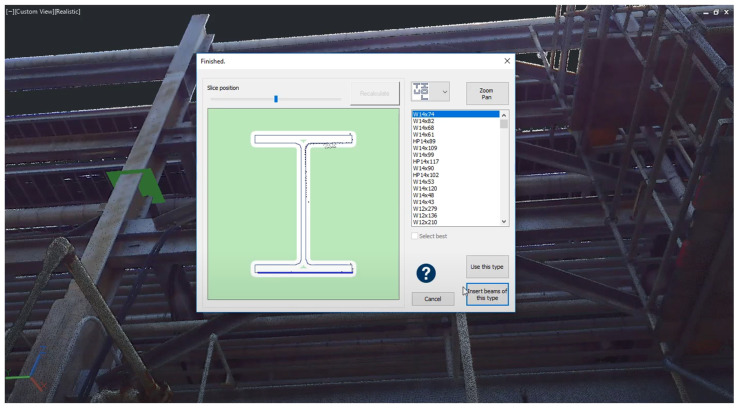
Automatic profile fitting in “Faro as-built”. Source [22].

Another approach for the detection of piping elements implemented in commercial software is an iterative semi-manual object detection approach. This approach exploits the fact that most piping elements are connected together and form continuous pipe runs. For example, “Faro as-built” uses a pipe or the end of a fitting, as well as its orientation, as the prior knowledge for the position of the next element. It automatically analyses PCDs at the suppositional location of the next element, automatically models the next element In the piping network, and fits a model from the catalogue of elements. The user verifies and adjusts the parameters of generated models to improve object identification. This process reduces manual effort but still requires keeping a human in the loop. In contrast to the “EdgeWise” approach, this method aims to provide exact specifications for each generated element. Objects with extruding geometry, such as beams and columns of various profiles, are not automatically generated by the existing software. For example, generating an I-beam in “Faro as-built” requires a user to specify the rough position of the object itself or a profile plane. Then, the software extracts the profile of the object and models it by using a catalogue of elements (Figure 6). Although it requires picking one to three points, these points can be manually selected for every object in the PCD. Lastly, software such as “EdgeWise” can automatically model structural elements, such as columns, if they are located on a regular grid after one of them is modelled.

**Figure 7:**
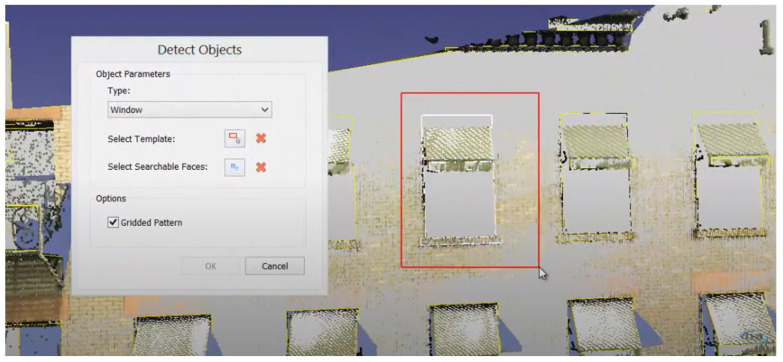
Template matching for object detection in “EdgeWise”. Red box shows selection of a template. Source [23].

Template matching is another approach for detecting and generating objects located on walls, such as doors and windows. For example, “EdgeWise” automatically detects a point cloud template in the limited part of the input (Figure 7). The search space is limited to a wall to ensure the computational feasibility of the search and practically limits the search along a plane with the known orientation of the template or just a small input. This does not require manual work for each object in the PCD and allows the scaling of the number of objects in the input.

Overall, commercial software for generating building geometry from a PCD can significantly reduce manual effort. The software can automatically detect primitive shapes and combine them into objects with relatively simple shapes, such as walls. Table 1 summarises existing software’s capabilities with respect to the automatic generation of the most frequently used object types in buildings from PCDs. However, there is still a substantial amount of manual work required. The user must verify every object detected automatically, manually adjust details (such as the boundaries of objects), and generate objects that were not detected, which is time-consuming.

### 2.2. Maintenance of Digital Twins

**Figure 8:**
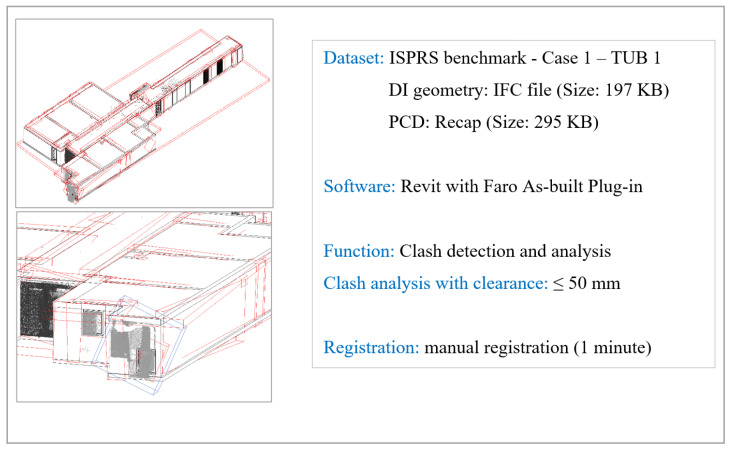
One example of the result for clash detection in Revit with the “Faro as-built” Plug-in.

The maintenance of gDTs requires updating product information by comparing as-is PCDs with previous statuses (e.g., as-designed models, previous as-built models). We summarise the three main steps to maintain gDT and elaborate on each of them based on the scan-vs-BIM system developed by Bosché et al. [24]: (1) DI geometry to spatial and visual data (SVD) registration; (2) SVD-vs-DI object instance detection; and (3) object instance geometry reconstruction. The first step ensures that the DI file (e.g., IFC file) and the as-built SVDs can be registered into a common global coordinate system. SVD refers to spatial data, namely PCD, and visual data, namely images and videos. Some current commercialised software are able to achieve coarse registration (e.g., Tekla Structure and Trimble RealWorks). The user needs to manually find at least three corresponding points both in the PCD and the DI geometry, then the software automatically calculates the transition and rotation matrix to register the PCD against the DI. Another way to achieve PCD to DI geometry registration is to manually adjust origins and axes to make coordinate systems the same. Images can also be registered by simulating camera poses in the 3D DI geometry to capture 2D pictures. This requires knowing the camera’s intrinsic and extrinsic parameters. The purpose of the first step is to facilitate the object instance detection as the second step between the DI-borne geometry and the as-built status during the construction stage.The second step contains PCD-vs-DI object instance detection and image-based object instance detection. This involves detecting object instances in input PCDs or images guided by DI. The output of this step is the extracted labelled point cluster corresponding to the designated object instance. To the best of our knowledge, no commercial software can automatically detect as-built object instances and match them with as-designed models in the SVD-vs-DI environment. Some software can perform clash detection between PCD and DI geometry, but they cannot focus on the instance level in order to detect and extract the whole individual component in the PCD. We carried out clash detection to verify whether it could be used to obtain point clusters of individual objects presented in the PCD. We used “Faro as-built” for Revit on the ISPRS benchmark TUB1 dataset [25] with the upper range clearance 50 mm. It took 22 min to complete clash detection on a computer (Processor: AMD Ryzen 5 5600X 6-Core Processor; RAM: 32 GB; GPU: AMD Radeon RX 6800). The result of 87 clashed elements contains over 90% unnecessary collisions, such as the noisy points of a part of an object instance (Figure 8). Therefore, clash detection cannot be directly used to match object instances for updating building gDT. Overall, the output data (e.g., extracted point cloud cluster) of the second step can be used to capture the 3D geometry of object instances in the third step.

The third step is to convert the extracted point cluster as low-level digitised 3D data into high-level volumetric 3D formats (e.g., 3D surface mesh). To the best of our knowledge, no software is capable of automatically capturing and recording the 3D geometry of as-built object instances from the PCDs with the help of DI. By contrast, two commercial solutions named OpenSpace and Buildots can measure construction progress by capturing images with a hat-mounted 360° camera. The image data stream is then compared with the expected progress from the DI to update the progress situation. Buildots can also evaluate visual quality by automatically detecting misalignment between images and DI. Nevertheless, these two solutions only rely on visual inspections to detect quality-related issues; they cannot update building gDT in a 3D view. The updated 3D geometry is essential for evaluating spatial quality during construction. Additionally, some software can automatically extract or generate limited object classes only from the PCD without being guided by the DI. For example, EdgeWise can automatically detect and generate cylindrical pipe segments, round joints (e.g., elbows, reducers), and rectangular duct segments without any manual effort. However, it is designed for PCD-to-BIM rather than PCD-vs-DI. It cannot help distinguish which as-built object instance belongs to which DI instance. Overall, to the best of our knowledge, there is no state-of-practice solution that can automatically keep building DT geometry updated based on the DI.

## 3. State of Research

This section explores state of the art in research in building geometry digitisation and the areas of computer vision relevant to gDT construction and maintenance for buildings. This includes space and object detection, which directly generates spaces and objects from PCDs; object matching and comparison, which allows the comparison of PCD with as-designed models; and image and point cloud segmentation, which may help with processing images and PCDs by enriching them with class labels. The construction of a gDT from a PCD is covered by the space and object detection methods and does not rely on reference geometry (i.e., previous as-built geometry or DI). The maintenance of gDT requires additional subsequent stages, namely matching objects from the PCD to objects from the reference geometry and comparing these objects with DI.

The decomposition of a PCD into a set of objects for buildings can be generally categorised into bottom-up and top-down approaches. Buildings are complex hierarchical structures consisting of floors, rooms, and hallways, with various objects inside and between spaces. The bottom-up methods start by detecting local features and composing them together to form objects, their combinations, spaces, floors, etc. The following subsection (Section 3.1) overviews bottom-up methods for object detection. Top-down methods start by detecting high-level and high-scale features and gradually decomposing them into smaller objects. Top-down methods for the digitisation of geometry for buildings are based on layout reconstruction (floor and space detection) methods because a layout is a high-level feature in a building. They then detect objects within spaces based on the information about spaces. Section 3.2 will review layout reconstruction methods for top-down geometry digitisation.

### 3.1. Object Detection

Object detection in a PCD refers to finding a particular object instance, including its location, in a PCD comprised of multiple object instances. This section discusses object detection methods for objects in buildings and related areas in computer science research, beginning with classic model-driven computer vision algorithms that represent deterministic methods for object detection and ending with data-driven deep learning methods for object detection that are useful for the generation and maintenance of gDTs. Object detection is relevant to the generation of the gDTs of buildings and to the second step of the three-step approach mentioned in Section 2.2.

#### 3.1.1. Classic Computer Vision Algorithms

Classical computer vision algorithms (also referred to as non-machine learning (ML) methods) for object detection are based on deterministic procedures for data analysis, such as primitive shape fitting and statistical analysis. The bottom-up methods for object detection in buildings are based on detecting and combining low-level features, such as planar or cylindrical patches, also referred to as primitive shapes. The basis for object generation methods are primitive shape detection methods, namely RANSAC [26], Hough transform [27], or region growing methods and their variations. These methods are designed to find shape parameters (or any hypothesis about data) in a given data or point clusters of individual geometric shapes; therefore, they can be applied to find shapes in PCDs with multiple shapes and fit parametric models into PCDs of a particular object. We refer readers to [28] for the review of RANSAC and its variations.

RANSAC-based methods can be applied for object detection during gDT generation and maintenance. For example, researchers use them to detect planar patches for wall generation [29,30]. These methods have also been used to detect pipes [31], cast-in-place footing detection [32], and cable trays and ventilation ducts [31], among other examples. These methods have also been used to verify the quality of precast concrete objects, such as walls and slabs, during construction [33,34]. After primitive shapes are extracted, rule-based methods can be used to adjust object dimensions, as shown in [35], where authors extended walls to remove gaps on corners. Zhang et al. [36] employed decision trees to compose geometric primitives together to form objects with more complex shapes.

Hough transform finds shape parameters to best describe the provide data for object generation from PCD. Researchers have shown how this method could be applied to detect pipes and other cylindrical elements [37,38,39]. It can also be used to estimate shape parameters from a PCD with the prior shape knowledge from the corresponding DI [40]. The applications of RANSAC and Hough transform are limited to object detection during the generation and maintenance of gDTs. Both methods are computationally expensive due to large inputs and inputs with multiple shape instances. They both require tolerance thresholds, which depend on data quality, the size of objects, and scene composition. They are also sensitive to clutter and occlusion and limited in the variety of shapes they can detect, working well for primitive shapes only. These methods fail if a notable amount of clutter is present in the PCD. Overall, these methods can successfully detect planar, cylindrical, or spherical objects in relatively small PCDs with small occlusions.

Region growing algorithms can also detect planar, cylindrical, and spherical objects in PCDs. These methods are based on the fact that the abovementioned shapes have homogeneous local curvatures throughout. This allows the selection of a point and the growing of a region by adding all neighbouring points with similar curvatures or normal orientations. As a result, such a method segments a PCD into clusters that represent objects, such as walls, slabs, pipes, ducts, elbows, or their parts [41,42,43,44]. These methods also allow the detection of objects from a reference model in PCD, when the object position deviates from reference geometry [45]. However, region growing methods are sensitive to noise because they greatly influence local parameters estimation, such as normal and curvature parameters. In addition, they tend to oversegment the PCD.

Other methods aim to reduce the problem into a 2D domain in order to simplify the object detection process. For example, Wang et al. [46] proposed slicing the input into multiple slices orthogonal to the X and Y axes and searching for the profiles of extruded objects or objects with non-trivial shapes. They showed how to use this method to detect pipes, beams and various extruded objects and machinery using template matching in 2D. However, the proposed method requires objects to be oriented parallel to the main axes only, limiting its applicability.

#### 3.1.2. Deep Learning for Shape Detection

Unlike classical computer vision approaches, data-driven methods for shape detection, such as deep learning methods, extract domain knowledge from labelled data provided to a model beforehand. These methods do not require specifying explicit knowledge in their design; therefore, they can be extended to multiple object types in multiple environments. Deep neural networks are used to classify individual points in PCDs, infer bounding boxes of objects, or classify point clusters. These applications can play a vital role in the geometry digitisation of buildings from PCD.

Bounding box prediction enables the detection of an object to reduce the complexity of generating its geometry. For example, Xu et al. [47,48] applied bounding box regression on indoor scenes on S3DIS [49] and SUNCG [50]. After a bounding box is determined for an object, this object can be modelled from a point cloud inside the bounding box. However, a bounding box may contain points from other closely located objects or if the object is not convex. This requires the further filtering of points irrelevant to the object. A point cluster of the object can be obtained by shape fitting using the above-mentioned methods, such as RANSAC or Hough transform, if the shape is known. The alternative approach is to obtain the point clusters of individual objects directly from the input. Thus, the instance segmentation of a PCD of a building will directly produce information required to generate the final geometry of objects.

The instance segmentation of PCD separates the input into individual point clusters, each representing a single object with its class. A point cluster can then be tessellated to obtain the geometric representation of each object. Park and Cho [51] applied class instance segmentation to the built environment in order to digitise the geometry of buildings, but they require the knowledge of the materials of objects as extra supervision to improve the performance. Mean precisions and recalls represent precisions and recalls averaged over all classes, where a positive detection is recorded if their intersection over union (IoU) exceeds 50%. The best models, such as those of Chen et al. [52], Jiang et al. [53], Liang et al. [54], Vu et al. [55], and Zhong et al. [56], score about 70% of precision and recall on S3DIS dataset, which means that only 70% of objects are detected, given that the intersection between the detected and the ground truth is at least 50% of their union, which declines if the IoU threshold is larger. It is worth noting that high recall is preferable over high precision because it is easier to delete an irrelevant cluster than to model it from scratch. Overall, it is not enough to automatically produce point clusters or meshes of individual objects from point clouds because it leaves 30% of objects to be processed manually and the further adjustment of point clusters to make IoU with a ground truth closer to 100%.

Object detection methods can also benefit from the 2D image analysis domain by adopting convolutional neural network (CNN) models. Czerniawski and Leite [57] applied DeepLab [58] architecture to automatically segment RGB-D (colour and depth) images into 13 building object classes, including windows, floors, stairs, walls, elevators, ducts, columns, ceilings, diffusers, doors, plumbing, furniture, and conduits. The experimental result shows that the average intersection of union (IoU) is 0.50. Narazaki et al. [59] applied a fully convolutional network (FCN) to detect bridge components. FCN is also used to detect building changes from remote sensing images with 88.86% of IoU from the experimental results [60]. Yeum et al. [61] applied AlexNet [62] to detect the welded joints of a highway sign truss structure. Liang [63] used the faster R-CNN (regions with CNN features) [64] to detect bridge components, while Kufuor et al. [65] used it to detect MEP components, including sockets, switches, and radiators, by training both RGB 360 and standard images. Overall, these methods based on R-CNN can achieve high detection accuracy (over 90%) because of the greater availability of labelled data for training. Pan et al. [66] used image sequences to detect small objects, such as light fixtures, room signs, etc., and aligned a videogrammetric PCD to gDT to enrich it with these objects.

Environments with limited labelled data available for training deep neural networks can benefit from synthesising labelled data from the 3D models of buildings. Studies, such as Nikolenko [67] and Tremblay et al. [68], showed that synthetic data, such as PCDs sampled from 3D models, can assist in overcoming the lack of real-world data. Researchers compared strategies for training models when real-world ground truth data were limited [69]. They claimed that neural networks trained on a large amount of synthetic data and real data (20% of the available real dataset) achieved worse performance—roughly 10% lower than a trained model on the entire real dataset. On the other hand, the model trained on mixed data achieved significantly better performance (~20% higher) than a model trained only on the real part of the same dataset. These experiments highlighted that artificial data could bridge the gap when a massive amount of real data are unavailable.

### 3.2. Layout Reconstruction

We define a building layout to be a space decomposition which has two levels: floors and rooms, halls, and other spaces. Layout reconstruction the process of generating a building layout based on its as-is PCD and it is a basis for top-down approaches. The first step is to decompose a PCD into point clusters of individual floors. Huber et al. [70] presented a simple floor detection method based on projecting a PCD onto the *Z*-axis and localising density peaks there. The method is based on the fact that floors and ceilings are large horizontal objects and the height of all points of a floor or ceiling has the same Z coordinate. The height of peaks and the knowledge of the approximate width of slabs and floor height allow the identification of each floor and ceiling. Scanners provide information about gravity direction and align the *Z*-axis of PCD. A similar idea can be applied to detect rooms inside the PCDs of individual floors. This approach is limited to horizontal floors only. It cannot successfully detect floors and ceilings if they are tilted or non-flat (e.g., variations in the hight of a floor).

Room boundaries can then be detected in the high-density areas of the X–Y projection of the PCD of a floor. Filtering points by keeping only points with normal perpendicular to the *Z*-axis improves the performance of the method because it keeps only points on vertical surfaces while discarding others. The wall occupancy information can then be used to compute the floorplan for the PCD. Alternatively, one could use the information on empty parts on the X–Y plane to infer a floor plan. However, this method assumes that walls would be the only major visible vertical structures in a building, which does not hold if there are other vertical objects, such as cabinets, or if walls are significantly occluded.

Macher et al. [71] proposed identifying rooms on a storey by computing a discrete occupancy map for the horizontal slice containing the ceiling. This method is based on the idea that rooms and hallways are disjointed at the ceiling because transitions between them, such as doors, have a smaller height than the ceiling itself. This assumption allows the segmentation of spaces by joining neighbouring occupied parts together. Afterwards, a 2D room layout can be refined by computing alpha shapes to reduce the influence of clutter and occlusions. This output allows the splitting of the input PCD of multiple rooms into multiple PCDs of single rooms.

Alternatively, a room arrangement can be detected by optimising the wall-space layout based on the information on potential wall location. For example, Ochmann et al. [72] projected points onto the X–Y plane and detected lines there. They then split space based on the faces spanned by lines and marked surfaces as belonging to the same space with the co-visibility information from the sensor (essentially whether points in the region are from the same scan). However, this requires extra input on top of the PCD and limits the applications of the method. They also marked line segments as potential wall locations if two lines nearly parallel lines were within wall thickness. Finally, they reformulated the space layout problem as an optimisation problem of mapping faces to rooms and classified line segments into walls and non-walls. They then used integer linear programming (ILP) to solve this optimisation problem and produced a space layout with walls. A similar approach was used in other papers, e.g., Ambruş et al. [73], Fang et al. [74], Han et al. [30], and Turner and Zakhor [75]; however, these approaches use RANSAC-based wall detection in 2D or 3D, which is not robust in the presence of noise, clutter, or occlusions. Additionally, this has similar limitations to other methods based on the X–Y projections mentioned above. Researchers also used data-driven approaches to provide information for ILP. Data-driven methods are less sensitive to noise, clutter, or occlusions but require labelled data. For example, Liu et al. [76] predicted room corners from RGB-D video and used ILP to obtain room layout. Similarly, Nauata and Furukawa [77] used a similar approach to detect buildings in outdoor areal images, while Chen et al. [41] used mask-RCNN to detect rooms on a PCD projection to a ground plane using panoramic RGB-D images.

Space decomposition can also be solved in 3D space. Tran et al. [78] proposed splitting the 3D space into cuboids based on the density peaks along each line. Then, they classified each cuboid and surface based on the PCD on the surface, manually added information on doors and windows, and iteratively merged them using the shape-grammar approach. Another method proposed is to detect empty spaces in the 3D PCD using the void-growing approach [79]. This method finds the empty regions of a PCD by growing a cuboid until it touches a wall or slab. In the first stage, it searches for vertical planes to produce room centre candidates (the authors split space through planes parallel to two main axes and obtained cube centres). Then, the method enlarges each cuboid in each direction until the points on the boundary of the cuboid surface occupy a significant part of the surface. In the final step of space detection, it discards thin cuboids (walls) and merges overlapping spaces to account for non-rectangular rooms, such as L-shaped rooms. Both of these methods allow the detection of rooms, hallways, and other spaces and their boundaries directly in 3D, but both are based on the Manhattan world assumption, which limits their application.

Overall, space detection methods reduce the complexity of object detection and provide information for wall location. The 2D floor plan can be used to split the PCD into multiple PCDs of individual rooms. This enables the parallel processing of multiple smaller PCDs for detecting objects located inside rooms. Additionally, it provides topological information about the building, which is necessary for generating gDT and developing its applications.

### 3.3. Object Matching

Object matching refers to matching a PCD cluster of an individual object with the corresponding DI geometry by comparing features extracted from both datasets. We separated methods into two classes: direct feature-based object matching and mesh-supported object matching. The former uses hand-crafted feature values (e.g., position, orientation, colour) extracted from object instances of a PCD and a DI, while the latter needs to transfer PCD or DI geometry to the format of mesh and then extracts the core vertices of object instances to support matching.

Feature-based object matching methods take advantage of the properties (e.g., position, orientation, scale, colour) of the object instance to support PCD detection. These methods extract features from a point cluster (a small set of PCD) and DI object, then compare them to decide whether they represent the same object instance. A method based on three features has been developed for detecting linear object instances (e.g., columns and beams) and surface object instances (e.g., slabs) for construction progress measurement [80]. The first feature is the Lalonde feature [81], which is a 3D vector for capturing lines and surfaces, the second feature is “orientation”, and the third feature is “continuity”. The method uses these features to remove points that are irrelevant to the object in the DI from a point cluster that represents this object. This method assumes that all object instances are located according to the DI. Similarly, a method based on five features has been proposed to match object instances using a “spatial context” matrix with features including length, scale, colour, orientation, and the number of connections with adjacent object instances [82]. This method was only tested for prefabricated pipe detection in an environment without any occlusion and clutter. A distribution-based 3D shape matching method has been developed by computing the probability distribution of the geometric properties for both PCD and the DI file [83,84]. The authors used 3D shape distribution (i.e., the probability distribution of the geometric properties sampled from segments in point cloud data and components modelled in a BIM) as the feature and calculated the shape similarity by Euclidean distance between the points of the components. However, this method requires a denoised PCD without any occlusion. Therefore, more generic methods need to be developed to match objects relying on features that are robust to shapes and spatial contexts. A mesh-supported method is therefore proposed.

The initial idea of the mesh-supported method applied the spin image to match object instances. The spin image was developed for 3D object instance matching [85,86]. It is a data-level shape descriptor representing the surface of the instance, which can be used to match tessellation surfaces between two datasets. A 2D array representation of a spin image is created by bilinear interpolation with projected distances [87]. The corresponding points in both as-built and as-designed tessellations can then be matched by comparing spin images. Kim et al. [88] also generated a tessellation from the sparse PCD to semi-automatically match object instances for quantity calculation and progress monitoring at the construction stage. This method also presents some limitations. First, the PCD is downsampled and approximated with a polygon tessellation, resulting in a loss of information initially contained in the PCD. Secondly, the method does not investigate all points (vertices) for matching object instances (typically only around 20~50%), which means that small or highly occluded object instances may be missed. Investigating all vertices may avoid this problem but leads to high computational complexity.

### 3.4. PCD to DI Comparison

PCD to DI comparison aims to detect whether object instances from a DI exist in the corresponding PCD. We illustrated two groups of methods for object comparison, namely point-to-point and point-to-surface comparison. These methods verify whether an object is present or not and cannot be used directly to extract entire points corresponding to the instance.

Point-to-point comparison methods generate points from the DI geometry and retrieve relevant points from the as-built PCD. They keep only those points from the as-built PCD that are close to points sampled from DI. Bosché and Haas [89] applied this method to automatically retrieve 3D object instances from PCD. They defined the retrieval rate 𝑅% as the ratio of the number of retrieved as-designed points to the total number of as-designed points. The threshold was set as 50% to assess the retrieval result, and the initial experiments on small-scale datasets (four columns and one slab, each within 18,000 points) presented robust results. Chen and Cho [90] also applied point-to-point comparison for automated PCD-vs-DI deviation detection for ductwork, columns, and beams. This method has been improved and applied for the detection of the main structural object classes [91,92] and mechanical object classes [40] to monitor progress at the construction stage. The experimental results show that the method can track the progress status of structural object instances, including floors, ceilings, beams, and columns, from the as-built PCD and the 4D DI combined with the schedule. By contrast, the experiment for mechanical object instance detection (round pipe segments and rectangular duct segments) fails to automatically detect object instances due to the higher false-negative or false-positive rates. Large spatial deviations between a PCD and a DI (e.g., 50mm or more) for mechanical objects cause higher false-negative rates. They also lead to higher false-positive rates in case part of one object instance is at the same location as another object instance in a PCD and a DI. This cannot be avoided in the experiments completely, even though the authors used the orientation of the surface’s normalas an additional rule. Turkan et al. [93] extended this method to track secondary and temporary object instances for progress monitoring in structural concrete work. The temporary structures include formwork, scaffolding, and shoring, while the secondary components include rebar.

Another method is point-to-surface comparison. It has also been used in the PCD-vs-DI object instance detection, including columns, walls, round pipe segments, and rectangular duct segments. This method calculates the ratio of the overlapping area between the segments extracted from the PCD and the object instance from the DI [83,84,94]. This method can also be used for deviation analysis between the as-built object instance and the DI [95]. Tran and Khoshelham [96] proposed an advanced surface coverage approach to make the method robust, which can theoretically detect arbitrary object classes. Euclidean distance is also used to determine the nearest point to the DI geometry surface, for instance, detection [97,98,99]. However, the limitation of all these methods is that they cannot recognise all points corresponding to the object instances when there are deviations between the PCD and DI geometry or in the PCD with high clutter. Additionally, the method requires the coverage ratio threshold to be manually set beforehand, and the method will fail if the deviation of the as-built position or orientation exceeds the ratio threshold. Currently, the existing research methods have only been tested on common object types, such as slabs, columns, or pipes, etc.

### 3.5. Summary

The construction of gDTs for buildings is based on object detection (Section 3.1) or layout reconstruction (Section 3.2) methods. The overall pipeline for object detection can rely on detecting large-scale features (top-down), low-level features (bottom-up), or a mixture of these (hybrid). The maintenance of gDTs also relies on DI as a reference for object detection. It can generally use one of the following two workflows: (1) use object detection methods to retrieve object instances from a PCD (Section 3.1 and Section 3.2) and then use object matching methods (Section 3.3) to match them to object instances from the corresponding DI or (2) use PCD to DI comparison methods (Section 3.4) to guide object instance retrieval from a PCD.

Table 2 summarises state-of-the-art research in object detection and layout reconstruction methods discussed in Section 3.1 and Section 3.2. Layout reconstruction methods provide spaces and objects that define building topology, namely slabs, walls, doors, and windows. In addition, they achieve two extra goals: reduce input for object detection methods and generate a hierarchical structure of a building. Object detection methods can generally be split into non-ML object detection methods and ML methods. The former group represents primarily deterministic methods that exploit explicit knowledge about object shapes, their contexts, environmental constraints, and others, but the assumptions that these methods make vary from method to method. One of the examples of such constraints in many object or shape detection methods is the Manhattan world assumption, which significantly limits these methods’ applicability. Their applicability to large or cluttered environments remains questionable, and their extensibility to alternative contexts is limited. In contrast, deep learning methods do not use explicit knowledge about buildings. They aim to capture this implicitly from labelled datasets during training. These models can be then used to directly infer point clusters of individual objects, infer minimal bounding boxes of objects to simplify object detection for methods discussed above, generate the layouts of buildings, or provide other forms of support. These methods can theoretically be extended to other object types or environments by providing labelled data, although we still lack practical evidence of this. However, these methods require large, labelled datasets, which are expensive to produce. Unfortunately, we have only a limited number of publicly available PCDs.

Few datasets are publicly available for digitising building geometry needs, namely S3DIS [49], which has the point clouds of an indoor environment with major structural elements labelled; ScanNet [100], which contains labelled data for furniture and some structural elements; SUN-RGBD [101], which has semantic segmentation and depth maps of indoor images; ISPRS [25], which has point clouds and corresponding IFC models; SUNCG [50], which consists of RGBD images of different models of rooms; Matterport 3D [102], which consists of multiple multi-level indoor scenes with instance annotations, among other things. It is still unclear how well deep learning methods work for environments different from those present in the labelled data provided to the model. It is also unclear how large the labelled dataset should be in order to generalise.

The maintenance of the gDT also includes object detection (discussed in Table 2), object matching, and PCD to DI comparison. The purpose of maintaining a gDT is to keep gDT dynamic and up to date in order to support progress monitoring and quality control by analysing discrepancies. The summary of the methods for object matching and DI-guided object detection is presented in Table 3. The methods for object matching require the as-built conditions to be roughly the same as the DI. For instance, the feature-based method and mesh-supported method require the same features extracted from two datasets before matching each other. They cannot match objects if there are moderate or distinct deviations in terms of shape, position, orientation, and scale between the DI and as-is instances. On the other hand, DI-guided object detection methods (point-to-point comparison and point-to-surface comparison) tolerate certain deviations in terms of shape, position, orientation, and scale between two datasets. They are able to retrieve object instances when there are minor discrepancies between two datasets. However, they cannot extract all points corresponding to the object instances, and the results highly depend on the retrieve rate setting.

**Table 2:** Summary of object instance detection methods for gDT generation and maintenance.

Detector	Methods	Existing Studies	Objects	Advantages	Core Limitations
Shape detector	Hough transform	[24,38]	Cylindrical pipe, round elbow	Easy to implement	Computational complexity; fails to detect occluded object instances
	RANSAC	[29,31,32,34,37,45]	Precast slab, wall, cast-in-place footing, cylindrical pipe, rectangular duct, cable tray, round column, round elbow	Easy to implement; robust to limited clutter and occlusions	Inefficient for large inputs or when a large number of objects are present; requires further processing; only robust for primitive shapes
	Region-growing	[43]	Walls, slabs, round pipes, round elbows, round columns	Scalable	Oversegmentation; a limited number of shapes; requires further processing; sensitivity to noise
	2D Slicing and projection	[46]	Round and rectangular pipes, columns, beams, heating and plumbing terminals	Objects of extrusion, arbitrary and complex shapes	Objects must be located along a limited number of axes
	Deep learning supervised PCD segmentation	[103]	All	Need only labelled data to generalise	Need a large set of labelled data to generalise
	Deep learning supervised on images	[11,57,61,65]	window, stairs, wall, elevator, duct, column, beam, slab, door, pipe, socket, switch, radiator	Theoretically extendable to arbitrary shapes	Requires alignment with 3D data to reflect spatial information
Layout generation	1D projections: Histograms of number of points	[70,78]	/	Easy to implement	Intolerant to clutter and occlusions
	2D projections: Floor-plan reconstruction	[71,72]	/	Structured, connected output	Rely explicitly on knowledge, design patterns; not extensible

## 4. Hudrokis Tree and Most Frequently Used Object Class Analyses

While there are thousands of different objects in a building, most of them are rarely used in gDTs. We hypothesise that only a few object classes are often modelled to support DT applications. Therefore, there is a need to identify the most frequent objects. Prioritising the most frequent object classes for the automation of geometry processing can significantly save time and reduce the cost of the construction and the maintenance of gDTs. In this section, we analysed the current building object classification standards and found the limitation that they do not support the generation and updating of building DTs from the geometric perspective. We then derived a new geometry-oriented building object class hierarchy to help classify objects from the shape perspective. Thereafter, we collected and analysed IFC models of various types of buildings and identified the most frequent object classes that appear in the gDTs of buildings. For the future directions of construction and the maintenance of gDTs, researchers are suggested to develop methods from the geometric perspective based on this new hierarchy and focus on these most frequently used objects to make methods more useful in the AECO industry.

### 4.1. Hudrokis Tree

We first needed to identify object classes from the geometric perspective that exist in a typical building before generating and updating their geometry in a gDT. There have been various building object classification systems developed by different institutions in the last sixty years, including SfB in Scandinavia (first edition in the 1950s), BSAB 96 in Sweden (1998), DBK in Denmark (2006), MasterFormat in the US (first edition in 1963), OmniClass in the US (first edition in 2006), UniFormat in the US (first edition in 1973), SFCA in the UK (first edition in 1961), and Uniclass in the UK (first edition in 1997) [104,105]. All these classification standards were developed to categorise building object instances, but each has its own criteria. We needed to conduct a detailed review of predominant standards to assess whether one of these can be employed to assist in generating and updating gDTs.

Four primary classification standards carried out by the UK and US institutes have been selected for a detailed review. These are Uniclass from the UK [106], the RICS (Royal Institution of Chartered Surveyors) classification from the UK [107], UniFormat from the US [108], and OmniClass from the US [109]. Table 4 summarises and compares the essential information and properties extracted from these four classification systems. The authors examined the latest versions of each standard and found that these standards focus on classifying elements by their functions or activities.

The database encompasses 15 tables centered on buildings, landscapes, civil, and process engineering, with specific focus on elements (Table 21), products (Table 23), services (Table 32). (Tables can be downloaded from [109].)

To conclude, current categorisations cannot be applied directly to facilitate DT geometry generation and updating because they are function-oriented and are designed to support activities during a building’s lifecycle. Therefore, we developed a new geometry-ended building object class hierarchy from a geometric perspective and named it the Hudrokis Tree (HT).

We made the following assumptions for generating the HT:

- We consider common building object types with explicit geometry on the IFC model level. We do not focus on the objects that are not integral parts of a building (e.g., the handle of the window, the door shaft, etc.) since these objects are rarely used in BIMs.
- We consider visible object types in buildings. Any inner objects, such as rebars and foundations, are not included in the Hudrokis Tree since they are out of its scope.

**Figure 9:**
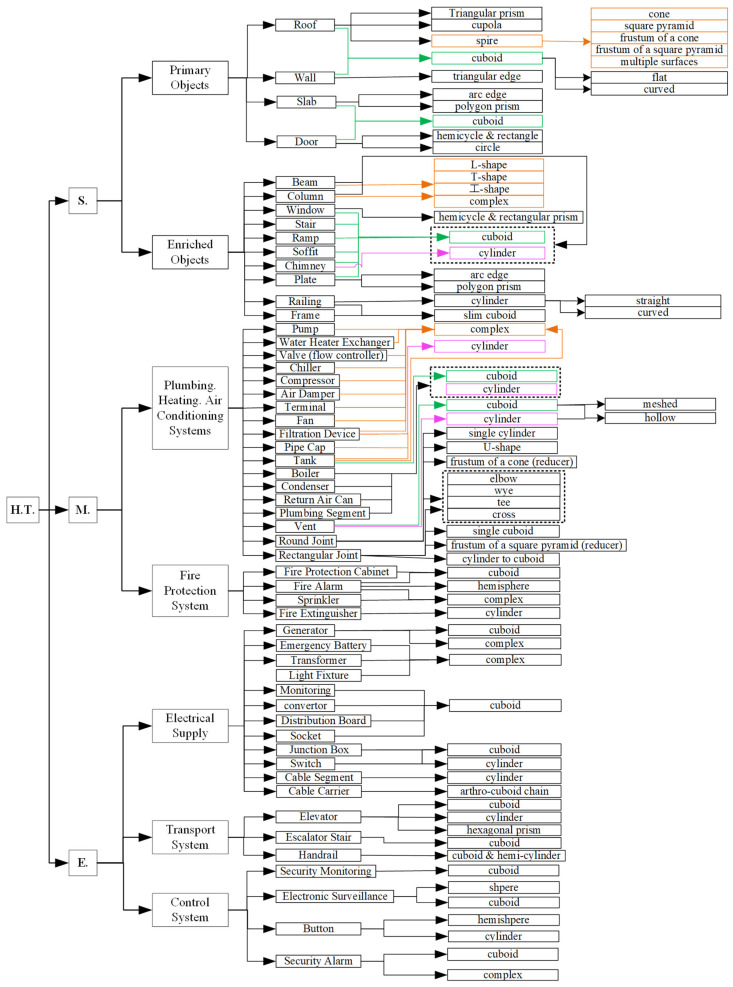
New geometry-based building object class hierarchy—Hudrokis Tree (H.T.).

We followed a top-down methodology to generate HT, starting from the functional categories and ending with the geometry level. The hierarchy of HT (Figure 9) comprises the major categories of building object types separated by their specific functions, followed by three levels that eventually deliver the geometry-oriented classification at the end leaves. Specifically, we divided building object classes into structural, mechanical, and electrical categories at the first level. Here, the structural category also includes all architectural components, such as partition walls, and non-structural objects in some definitions. The structural category contains four primary object classes and ten enriched object classes. The mechanical category includes eighteen object classes from plumbing, heating, and air conditioning systems and four object classes from the fire protection system. Similarly, the electrical category comprises twelve object classes from the electrical supply, three object classes from the transport system, and four object classes from the control system. The geometry of the majority of these classes is 3D cuboids or cylinders.

HT is different from existing classification standards since it is geometry-ended. Specifically, it merged certain object types with different functions but the same geometry into one object class. For instance, Uniclass and OmniClass categorise service systems into plumbing, heating, cooling, and ventilation functions. Each function contains a range of various pumps, tanks, pipes, ducts, drains, and other components sorted by their different roles [109,110]. The hierarchy of HT is instead based on geometry classes. We merged duct, piping, and drain segments into one segment class named the “plumbing segment” because all these object class geometries can only be a cuboid or a cylinder. Similarly, we integrated all types of mechanical connection components into two classes based on their geometry representations: round joints and rectangular joints. Here, “round” and “rectangular” refer to the cross-sections of shapes. Various shape-based classes, including elbow-, wye-, T-, cross-, and U-shaped, are then listed on the next level in order to refine the specific geometry classes of round joints and rectangular joints.

HT meets two core characteristics that make it more applicable for constructing and maintaining gDTs than the existing classification standards: (1) it contains all common visible building object classes of interest to the design, construction, and operation stages. Any non-visible object types, such as foundations, piles, and ground beams are not included in the HT. (2) It is a geometry-ended classification standard. The end leaves of this new tree contain the most common existing shapes for each object class. For example, the common 3D geometry of walls shown in the Hudrokis Tree contains a flat cuboid, curved cuboid, and triangular edge, which always exit in traditional British houses. This geometry-ended classification guides researchers to focus on the common shapes in order to develop solutions for the generation and updating of gDT. Specifically, the tree can be used as prior knowledge to support rule-based geometry construction and maintenance or to facilitate the collection of a deep-learning-based training dataset. We regard the Hudrokis Tree as a comprehensive and valuable classification system for constructing and maintaining gDTs during the building’s lifecycle. It is a prerequisite for ranking the top frequent object classes in a typical building in the next section.

### 4.2. Frequency Analysis

The next step in defining building structure is identifying the average fractions of each component. It is necessary to build the order of object types for automation. The frequency analysis provided insight into what object classes professionals usually generate and update, and therefore, what is usually requested by the industry. The priority would be given to more frequent objects since higher frequency implies higher effort in performing asset geometry generation and updating. The frequency analysis aims to identify which object classes are more frequent than others, not to identify object frequencies with high precision.

We gathered a dataset of 24 sufficiently complete “Industry Foundation Classes” (IFC) models of buildings on the design, construction, and operation stages for this frequency analysis. Table 5 shows the distribution of the number of objects in the dataset. The sources of data were open repositories [111], industry partners (Trimble), and the university’s resources (Department of Engineering, University of Cambridge). The models represented various types of buildings, including office buildings, university buildings, hospitals, and residential buildings.

The analysis aimed to obtain the number of frequencies of each object class in the dataset. Each IFC object representing a physical item of every IFC model was added to the list of object instances. Objects in this list were then mapped into the classification introduced previously. This mapping was carried out based on either the object’s type and parameters in the IFC schema or its textual description. IFC type was used for the type mapping if it allowed the unambiguous identification of the object type in the proposed classification (for example, “IfcWall” is mapped to “Wall”). However, it was not always possible to uniquely map based on the IFC type; therefore, IFC parameters, such as the object’s geometry (for example, to separate round and rectangular “IfcDuctSegment”s), were taken into account in this case. The classification of an object can be attempted based on its textual description provided by a modeller by searching for keywords if previous rules failed to identify its type. Objects that could not be processed based on the strategies above were considered invalid or ambiguous and inspected manually.

This procedure produced a table of counts for each object class and each building model (i.e., how many objects of the type “T” is in building “B”). However, some models from the dataset missed objects from some level-one categories, namely structural, mechanical, or electrical. There are nine models with electrical objects, eighteen models with mechanical objects, and twenty-four models with structural objects. Therefore, the counts of missing categories should have been compensated for in unmodelled categories. This was built based on the following assumptions:

- All valid objects are from one of three categories: structural, mechanical, or electrical.
- A category is considered to be modelled entirely for a file if at least one object within the category in the file is modelled.
- The distribution of objects’ fractions within a category is multinomial and the same for all models.
- The distribution of category fractions is multinomial and the same for all models.

The first step in compensating for missing counts was to estimate fractions for each level one category. The key observation for calculating category frequencies was that all models have either electrical objects missing or electrical and mechanical objects missing. This allowed the estimation of these fractions consequently. In the latter equations, $S_{i},\;M_{i},\;E_{i}\;$ denote the total number of structural, mechanical, and electrical objects in the i-th model, respectively; $p_{S},\;p_{M},\;p_{E}\;$ denote the fraction of structural, mechanical, and electrical objects in the dataset (i.e., the number of structural, mechanical, and electrical objects in the dataset divided by the total number of objects in the dataset given that all intended objects are modelled), respectively; and Equation (1) shows the indicator function. Counts for the electrical category were computed at this stage by summing the counts of all electrical objects across all complete models and then dividing by the total number of objects in these models (Equation (2)). The next step was to estimate the fraction of mechanical objects among structural and mechanical objects. This was performed similarly by considering all files that have objects of these two categories. After that, the fraction of mechanical objects was computed by accounting for a fraction of the electrical category (Equation (3)). Lastly, the fraction of structural objects can be computed as a remaining part (Equation (4)).

(1) $$\delta \left(x\right)=\left\{\begin{array}{c} 1,\;if\;x\;\neq 0\\ 0,\;if\;x=0 \end{array}\right.\;$$

(2) $$p_{E}=\frac{\sum_{i}^{}E_{i}}{\sum_{i}^{}\delta \left(E_{i}\right)(E_{i}+S_{i}+M_{i})}$$

(3) $$p_{M}=\frac{\sum_{i}^{}M_{i}}{\sum_{i}^{}\delta \left(M_{i}\right)\left(S_{i}+M_{i}\right)}\times \left(1-p_{E}\right)$$

(4) $$p_{S}=1-p_{M}-p_{E}$$

We adjusted the counts of objects by introducing “missing” objects for missing categories after these fractions were identified. The category count was multiplied by the percentage of the object type within the category to convert the “missing” counts of categories into the “missing” counts of individual objects. These percentages were determined empirically from the dataset by summing the object type counts for all files and dividing them by category size. This adjustment neglected the effect of unmodelled categories in some of the presented models.

The frequency analysis of the tree in Figure 9 is based on 24 IFC models that include 105 k objects. The dataset of the 24 IFC models consisted of 105,000 objects (with more than 100,000 real objects and about 5000 “missing” objects). The empirical fractions of the object categories were 35.29% for structural, 58.22% for mechanical, and 6.49% for electrical objects. The top 10 most frequent objects are listed below (Table 6).

Table 7 lists the top ten structural objects. The statistical analysis showed that the top six structural elements accounted for more than 96% of structural elements for buildings. These were walls, beams, columns, slabs, doors, and windows. Others made up less than 4%. Table 8 presents the fractions of the top 10 objects of the mechanical objects class. The top two objects, namely round pipe segments and round joints, constituted about 60% of all mechanical objects. The following five object types (plumbing and heating terminals, round cylindrical connections, rectangular duct segments, round Ts, and reducers) increased the coverage to 88% of all mechanical objects. Lastly, Table 9 shows the frequencies of the top eight electrical object types. Here, the top two object types (light fixtures and alarms) represented 98% of all electrical objects in the dataset.

The combination of the above-mentioned top object types from each category covered a significant portion of objects within a building. The top six structural, the top two mechanical, and the top two electrical objects represented 75% of all objects in a building on average. Therefore, the automation of the geometry generation of these ten object types was prioritised over other objects. The overall coverage of objects in a building rose to 92% by replacing the top two mechanical object types with the top seven. 

## 5. Discussion

Space and object detection is a core step for the construction and maintenance of gDTs. Commercial software available today assists object detection by detecting geometric primitives, such as cylinders for pipes and columns or planes for slabs, walls, and other planar objects. Alternatively, the software can refine parameters for object geometry based on the point clusters of individual objects, which works well in the presence of a catalogue of objects. However, it is unable to do both at the same time since the latter option is sensitive to the quality of point cluster generation.

Approaches for space detection, object detection, object matching, and PCD to DI comparison are discussed in Section 3.5 and summarised in Table 2 and Table 3. Section 4.2 then analysed the IFC models of multiple buildings and identified the most frequent object classes, namely walls, slabs, beams, columns, doors, windows, round pipe segments, round elbows, light fixtures, alarms, plumbing and heating terminals, and various pipe fittings, which cover up to 90% of objects on average. Most of these object classes are considered in commercial software or state-of-the-art research for automatic or semi-automatic construction or maintenance.

- **Walls, Slabs:** Many software programmes, such as PointFuse and EdgeWise, can automatically detect walls and slabs as planar surfaces and combine them together to form volumetric models of their geometry. Many research papers also focus on wall and slab detection for the construction and maintenance of gDTs. The authors used RANSAC, Hough transform, region growing-based methods, or feature-based methods to detect large planar patches, e.g., [24,29,30,43,84]. Additionally, many room detection methods produce walls as objects separating rooms, e.g., [70,71,72,78]. However, these methods remain sensitive to noise, clutter, and occlusions. In addition, some papers used point-to-point/surface methods to monitor the progress of slabs [91,92].
- **Beams, Columns:** Software does not provide automatic tools to detect beams and columns from a PCD but can automatically determine profile parameters and length once a part of an object or its approximate location is detected, for example, “Faro as-built” or Leica. A few research papers focus on beams and columns for automatic detection, such as [46], but this approach is limited due to the assumptions for the direction of the axis. The automation of round column detection is more mature since they have simpler geometry (cylinder). This class of object can be detected using RANSAC, Hough transform, region growing-based methods, or feature-based object matching [24,38,80,82,84] with the limitations of these methods mentioned above.
- **Doors, Windows:** Software such as EdgeWise can detect doors and windows via template matching on surfaces; however, the search space for this approach is limited. Research proposes methods for door and window detection on walls, using colour inconsistency for closed doors [112] or searching for doors in voids on walls for opened doors [44]. Additionally, current research methods cannot deal with transparent windows, doors, and walls.
- **Pipe Segments, Elbows, and other Pipe Fittings:** Round pipes have a cylindrical shape and can be automatically detected by many types of software, such as EdgeWise and many RANSAC, Hough transform, region growing methods, feature-based methods, and point-to-point/surface methods, e.g., [24,43,46,83,94,96]. Pipe fittings, on the other hand, are detected on the ends of piping segments. Some software detects pipes and fittings semi-automatically using iterative pipe runs.
- **Light Fixtures, Alarms, Plumbing, and Heating Terminals:** These objects are usually smaller and have less constrained shapes. No software is developed to detect these objects. Some software allows template matching but is limited or computationally inefficient. In addition, the mesh-supported method can detect small and shape-arbitrary objects by matching corresponding vertices in the scan-vs-BIM system [86,88] but can cause the loss of information due to downsampling PCD and cannot match highly occluded object instances.

RANSAC- and Hough transform-based methods are robust to noise to some extent but sensitive to the presence of clutter or occlusions. Region growing methods, on the other hand, are less sensitive to clutter and occlusions but sensitive to noise. These are also scalable to large PCDs, unlike RANSAC and Hough Transform, which become computationally inefficient with large inputs.

Deep learning methods can be generally applied to any object classes, given that there are enough labelled data to train neural networks. Many deep learning methods have been tested in bounding box regression or the PCD semantic and instance segmentation of walls, slabs, doors, windows, columns, beams, or pipes in indoor PCDs, see, e.g., [47,48,65]. These methods can also be applied to the remaining classes of objects; however, there is no scientific evidence of their success. Generally, deep learning methods tend to perform better on large objects, such as walls and slabs, and perform worse on small objects, such as pipe fittings and plumbing terminals.

The state-of-practice software simplifies the generation of the most frequent object types in buildings to some extent. The software can automatically detect primitive shapes, such as planar patches and cylinders in PCDs, and provide a user interface to generate objects and connect them to create DTs. These tools significantly reduce the manual effort necessary for digitising the geometry of existing assets from PCDs.

However, the generation of each object still requires some human intervention. Objects with planar surfaces require the manual adjustments of dimension sizes. For example, automatically generated walls and pipes in “EdgeWise” require a manual extension or shortening along the length. Automatic pipe fitting generation in this software considers only pipe endings and does not account for the PCD itself. It results in objects with the wrong parameters (e.g., elbows with the wrong radius). Semi-automatic pipe run detection is implemented in “Faro”, which tries to automatically fit the next network element and requires manual involvement for each object. The same holds for the detection of steel structures.

**Figure 10:**
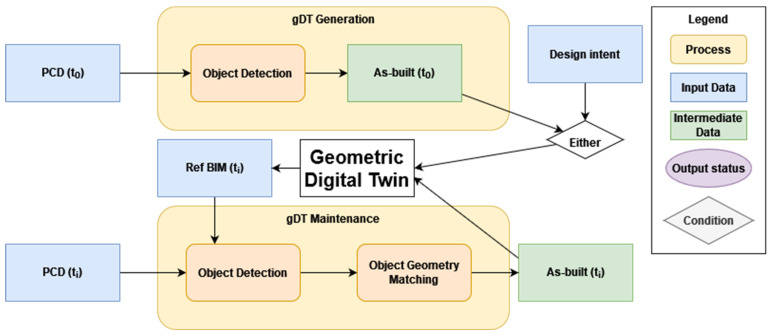
Generation and maintenance of gDTs.

The state of research covers all most frequently used object classes identified in this paper except plumbing and heating terminals and some pipe/duct fittings. Although multiple methods can be applied to detect and generate these objects separately, the input should satisfy the assumptions of all methods used. Moreover, even if all assumptions hold, such approaches yields different levels of quality and details for different object classes and these objects are completely disconnected from each other. To the best of our knowledge, there is no end-to-end or unified pipeline either in practice or in research that enables constructing and maintaining gDTs. To this end, we incorporated some of the aforementioned methods and approaches into a framework (Figure 10) that supports the construction and maintenance of gDTs.

The framework incorporates the main steps for gDT construction and maintenance, provides a basis for the gDT lifecycle, and highlights the group of methods helpful for geometry digitisation. Depending on the stage of a physical twin lifecycle, a gDT originates through gDT generation from PCD for existing buildings without a gDT or as-built model or an as-designed model serves as a start for a gDT for buildings that are in the design or construction stages. Implementing this framework offers several benefits, such as a streamlined and standardized approach to gDT construction and maintenance, which reduces the need for customized solutions and the ability to generate gDTs at different levels of detail for different object classes. Additionally, our framework provides a practical solution for keeping up-to-date building geometric data, allowing users to benefit from it through various digital twin applications, such as predictive maintenance, energy management, and real-time monitoring.

### Gaps in Knowledge, Open Questions, and Future Research Directions

Based on the review of the methods for object detection, object matching, and object comparison methods, we identify the following challenges and gaps in knowledge that should be addressed in order to enable the efficient automation of gDT generation and maintenance from PCD:

- Most of the classical computer vision methods are evaluated on different private datasets, which complicates their comparison. Therefore, it is hard to identify which methods work best for different object types. Researchers should evaluate these methods on public datasets to make the fair comparison of methods easier.
- Existing non-ML methods are hardly generalisable for arbitrary buildings and objects. Although knowledge about buildings helps make non-ML methods less complex and more robust, they exploit specific assumptions that limit their practical applications. The presence of noise, clutter, and occlusions make the problem harder. The industry would benefit from more generic methods for object detection and matching. We believe that deep learning methods can play a vital role in developing generic methods. How can we apply deep-learning methods to help detecting objects, spaces, and relationships between them?
- Following the previous point, classic computer-vision methods cannot detect the majority of objects with non-primitive shapes, such as various pipe joints or light fixtures, in various environments. Many non-ML methods are designed to detect primitive shapes, such as cuboids, cylinders, and spheres. We believe that adopting deep learning methods can tackle this problem. How can we design deep-learning methods to robustly detect complex shapes in PCDs? How can we incorporate prior knowledge to guide these methods?
- Matching an object instance between the PCD and the reference model when its location in both domains deviates notably is still challenging. We can find similarities between individual objects, but we cannot always identify them unambiguously due to the highly repetitive ensembles of objects. Additionally, current research methods are only suitable for progress monitoring but not quality control, because they cannot extract all points corresponding to the instance; thus, the extracted result cannot be meshed and analysed for quality assessment. We believe that adopting unsupervised clustering methods with the support of DI and images could be a potential solution. Alternatively, anaylsing object composition can help to reduce the ambiguity of object instance matching. How can we analyse object composition by primitive shapes?
- Deep learning methods require enough labelled data that include different geometric objects, but the amount of available labelled 3D data for the built environment are limited. This hinders the full automation of geometry digitisation. In addition, most of the current methods are not generalised with existing public data. We believe that adopting synthetic data for training may overcome the limited labelled data. How much real-world labelled data do we need? How should we generate and use synthetic data to reduce the size of real-world labelled data?
- PCD acquisition techniques are still not fully capable of capturing transparent and specular objects, which are integral parts of buildings. We do not know how to capture this type of object. We believe PCD data are insufficient to detect these objects because the points on such surfaces are significantly mislocated or removed as noise. We believe that incorporating 2D image analysis into geometry digitisation should help to detect transparent and specular objects. How can we seamlessly incorporate 2D image analysis? How can we seamlessly correspond 3D and 2D data with each other?

## 6. Conclusions

This paper provides an overview of the gDTs of buildings and their construction and maintenance during the construction, operation, and management stages. We discussed most of the well-known software in the AECO industry and to what level they automate the geometry digitisation process. We highlighted the current state of research and other relevant areas of study and the main limitations of the current methods. As a result of the discussion, we created a categorisation of building’s objects from the perspective of the objects’ geometry. We collected IFC models of buildings and performed frequency analysis to identify the most frequent object types in buildings, which should be prioritised for the automation of their detection, matching, and comparison.

It is essential to acknowledge the limitations of the research. One of the limitations of this paper is that the frequency analysis focused only on the most frequently used building elements that can be prioritised for automation, which may not capture the full range of building elements and does not capture other aspects that may be important to particular applications. It aimed to capture the entire range of applications by averaging frequencies from the IFC models created for various applications. Furthermore, this review did not include an evaluation of the effectiveness or accuracy of the presented methods. Finally, this study focused on typical buildings, such as offices, hospitals, and residential buildings. Therefore, the applicability of the findings may not apply to buildings of a significantly different nature, for example, heritage buildings.

The automatic generation of gDT from PCD will reduce the costs associated with creating a DT, making it commercially viable. This offers a wide range of benefits during the building operation and renovation stages, such as energy performance optimization, improved information management, schedule optimization, and reduced building operation costs, among others. Additionally, maintaining a dynamic gDT during construction allows for progress monitoring and quality control, which can catch mistakes and delays earlier in the process, resulting in shorter schedule delays and smaller budget overruns. We believe that tackling the aforementioned challenges can significantly improve the automation of geometry generation and updating for buildings. This contributes to the reduction of gDT costs and unlocks the benefits of DTs for the existing buildings, such as information management, predictive maintenance, and optimisation for renovation. It will also enable automatic progress monitoring and quality control during the construction stage of buildings’ lifecycles. As a result, buildings will become cheaper in construction and operation. This will improve construction productivity and reduce prices in built environments. This entails reductions in housing prices, office space and commercial space rents, and the final prices of goods. Additionally, more efficient construction reduces the environmental impact of the industry.

## Figures and Tables

**Table 1:** Comparison of existing software for building gDT construction. The table depicts whether the software can model a specific object type and how.

	Round Pipe Segment	Wall	Round Elbow	Window	Beam	Door	PlumbingHeatingTerminal
EdgeWise (Building and Plant packages)	Automatic (cylinder extraction)	Semi-automate (few clicks to adjust profile through one slice)	Automatic (Autocompletion pipe runs)	Templates	Semi-automatic (grid pattern detection)	Templates	Manual
Faro as-built modeller	Semi-Automatic (Cylinder extraction and best pipe type fitting)	Manual	Semi-Automatic (autocompletion of pipe runs)	Manual	Semi-automatic (few clicks to adjust profile through one slice)	Manual	Manual
Pointfuse	Manual (Extract meshes)	Automatic (extract planar meshes, can classify planar meshes)	Manual	Manual	Manual	Semi-Automatic (Auto planar meshes)	Semi-Automatic (extract planar meshes, can classify planar meshes)
Scantobim.xyz	Semi-Automatic (click on pipe point → cylinder extracted → adjust manually)	Manual	Manual	No	No	No	No
InfiPoints	Semi-Automatic (Automatic cylinders)	No	Manual	No	Manual (automatic cylinders and plates, a few click selection to model beam)	No	No
PointCab	No	Semi-Automatic (Automatic planes)	No	No	No	No	No
Vision Lidar	No	Automatic (PCD semantic segmentation)	No	Automatic (PCD semantic segmentation)	No	Automatic (PCD semantic segmentation)	No
Leica CloudWorx and Cyclone Model	Semi-Automatic (specify one point in a pipe run → a pipe run with segments and elbows is extracted)	No	Semi-Automatic (specify one point in a pipe run → a pipe run with segments and elbows is extracted)	No	Semi-automatic (few clicks to detect a beam, then adjust it)	No	No
E3D Design	Semi-Automatic (Select point on pipe, select point on another segment then pipe segments and elbows are modelled)	No	No	No	No	No	No
Trimble RealWorks	Semi-Automatic (autoextract cylinders and pipe runs in isolated point-clouds)	No	Semi-Automatic (autoextract cylinders and pipe runs in isolated point-clouds)	No	Semi-Automatic (no information)	Manual	No
Smap3D	Semi-Automatic (automatic pipe detection, then manual adjustment and connection)	Manual (planar surfeces detection)	No	No	No	No	No
Tekla Structure	Manual	Semi-Automatic (planar shapes)	Manual	Manual	Semi-Automatic	Manual	Manual

**Table 3:** Summary of object instance matching and comparison methods for maintenance.

Categories	Methods	Existing Studies	Objects	Advantages	Core Limitations
PCD-vs-DI detector	Point-to-point comparison	[40,89,91,92,93]	Column, beam, slab, wall, cylindrical pipe, rectangular duct, formwork, scaffolding, shoring, rebar	Theoretically extendable to arbitrary shapes	Fails to detect instances with distinct deviation; sensitive to clutter/occlusion
	Feature-based method	[80,82,84]	Wall, ceiling, column, beam, slab, rectangular duct, cylindrical pipe, round elbow/reducer	Theoretically extendable to arbitrary shapes	As-built must be the same as as-designed; requires as-built without any occlusion and noise; fails to detect glass-made or curved planes
	Point-to-surface comparison	[84,94,96]	Column, wall, cylindrical pipe, round elbow, round reducer, rectangular duct	Theoretically extendable to arbitrary shapes	Cannot extract all points corresponding to the object instance
	Mesh-supported method	[86,88]	Cylindrical pipe, U-shape round joint, wye joint, cross joint, slab, wall, beam, column	Theoretically extendable to arbitrary shapes	Loss of PCD information when meshing; small and highly occluded instances may be missed

**Table 4:** Summary and comparison among four predominant building elemental classification systems.

Standard	Uniclass	SFCA (BCIS Elements)	UniFormat	OmniClass (OCCS)
**Latest Version**	Uniclass-2023	SFCA-2012	UniFormat-2010	OCCS-2012
**Function**	For design and construction stages, documentation, specification.	For Elemental cost plans, “Element” is defined as a physical building part with specific functions.	For the building lifecycle management, cost analysis.	For the building lifecycle information management.
**Attribute**	The database comprises 14 fundamental tables, with “Elements” categorizing key structural components, including walls and roofs in a building or edifice.	The hierarchical data structure consists of 14 primary classes, with the first six classes categorising the building elements into further 3 sub-levels,	The hierarchical data structure features 5 levels, encompassing major construction information categories distinguished by their functions, including shell and interiors.	The database encompasses 15 tables centered on buildings, landscapes, civil, and process engineering, with specific focus on elements (Table 21), products (Table 23), services (Table 32). (Tables can be downloaded from [109].)

**Table 5:** Size of models in the dataset.

Number of Objects	Number of Models
0–99	4
100–999	9
1000–9999	8
10,000+	3

**Table 6:** Top 10 object types. The fractions of particular object types among all objects.

Rank	Category	Component	Total	Fraction (%)
1	Mechanical	PipeSegment.Round	24,645	23.44
2	Structural	Wall	11,432	10.87
3	Mechanical	RoundJoint.Elbow	11,411	10.85
4	Structural	Beam	8224	7.82
5	Structural	Column	5284	5.03
6	Structural	Slab	5283	5.02
7	Electrical	LightFixture	4755	4.52
8	Mechanical	PlumbingHeatingTerminal	4253	4.05
9	Mechanical	RoundJoint.SingleCylinder	4088	3.89
10	Mechanical	PipeSegment.Rectangular	3617	3.44

**Table 7:** Top 10 structural objects. The fractions of particular object types among structural objects.

Rank	Component	Total	Fraction (%)
1	Wall	11,432	30.81
2	Beam	8224	22.16
3	Column	5284	14.24
4	Slab	5283	14.23
5	Door	2867	7.72
6	Window	2548	6.86
7	Railing	807	2.17
8	Stair	506	1.36
9	Roof	138	0.37
10	Ramp	15	0.04

**Table 8:** Top 10 mechanical objects. The fractions of particular object types among mechanical objects.

Rank	Component	Total	Fraction (%)
1	PipeSegment.Round	24,645	40.25
2	RoundJoint.Elbow	11,412	18.64
3	PlumbingHeatingTerminal	4253	6.94
4	RoundJoint.SingleCylinder	4088	6.67
5	PipeSegment.Rectangular	3617	5.9
6	RoundJoint.Tee	3341	5.45
7	RoundJoint.Reducer	2797	4.56
8	RectangularJoint.Elbow	1574	2.57
9	Sprinkler	1463	2.38
10	Joint.RectangularToRoundTransition	1042	1.7

**Table 9:** Top 8 electrical objects. The fractions of particular object types among electrical objects.

Rank	Component	Total	Fraction (%)
1	LightFixture	4756	69.67
2	Alarm	1911	27.99
3	CableSegment	113	1.65
4	CableCarrierSegment	20	0.29
5	JunctionBox	11	0.15
6	Switch	11	0.15
7	Convertor	3	0.03
8	DistributionBoard	1	0.01

## Data Availability

The data and models used in this study were provided by a third party and unavailable due to privacy.
